# Specific Internalisation of Gold Nanoparticles into Engineered Porous Protein Cages *via* Affinity Binding

**DOI:** 10.1371/journal.pone.0162848

**Published:** 2016-09-13

**Authors:** David Paramelle, Tao Peng, Paul Free, David G. Fernig, Sierin Lim, Nikodem Tomczak

**Affiliations:** 1 Institute of Materials Research and Engineering, A*STAR (Agency for Science, Technology and Research), Singapore, Singapore; 2 Division of Bioengineering, School of Chemical and Biomedical Engineering, Nanyang Technological University, Singapore, Singapore; 3 Department of Biochemistry, Institute of Integrative Biology, University of Liverpool, Liverpool, United Kingdom; 4 NTU-Northwestern Institute for Nanomedicine, Nanyang Technology University, Singapore, Singapore; Brandeis University, UNITED STATES

## Abstract

Porous protein cages are supramolecular protein self-assemblies presenting pores that allow the access of surrounding molecules and ions into their core in order to store and transport them in biological environments. Protein cages’ pores are attractive channels for the internalisation of inorganic nanoparticles and an alternative for the preparation of hybrid bioinspired nanoparticles. However, strategies based on nanoparticle transport through the pores are largely unexplored, due to the difficulty of tailoring nanoparticles that have diameters commensurate with the pores size and simultaneously displaying specific affinity to the cages’ core and low non-specific binding to the cages’ outer surface. We evaluated the specific internalisation of single small gold nanoparticles, 3.9 nm in diameter, into porous protein cages *via* affinity binding. The E2 protein cage derived from the *Geobacillus stearothermophilus* presents 12 pores, 6 nm in diameter, and an empty core of 13 nm in diameter. We engineered the E2 protein by site-directed mutagenesis with oligohistidine sequences exposing them into the cage’s core. Dynamic light scattering and electron microscopy analysis show that the structures of E2 protein cages mutated with bis- or penta-histidine sequences are well conserved. The surface of the gold nanoparticles was passivated with a self-assembled monolayer made of a mixture of short peptidols and thiolated alkane ethylene glycol ligands. Such monolayers are found to provide thin coatings preventing non-specific binding to proteins. Further functionalisation of the peptide coated gold nanoparticles with Ni^2+^ nitrilotriacetic moieties enabled the specific binding to oligohistidine tagged cages. The internalisation *via* affinity binding was evaluated by electron microscopy analysis. From the various mutations tested, only the penta-histidine mutated E2 protein cage showed repeatable and stable internalisation. The present work overcomes the limitations of currently available approaches and provides a new route to design tailored and well-controlled hybrid nanoparticles.

## Introduction

Protein cages and virus-like particles are highly structured and stable homo- and hetero-supramolecular protein self-assemblies.[1] All protein cages share three common structural features: an external surface, an inner surface or core, and a protein-protein interface. Recent advances in molecular biology and organic chemistry enable the modification of these features independently[2] using genetic mutations, e.g., to replace or introduce amino acid residues at desired locations, or by bioorthogonal conjugation[3] to add non-native functions, e.g., drugs, biomarkers or fluorescent dyes.[4] Such flexibility of modification with relevant features has enabled the utilisation of protein cages for a wide range of applications such as bioimaging and drug delivery.[4–8]

During the last two decades, materials scientists have intensively investigated the manipulation of protein cages for the encapsulation of single inorganic nanoparticles into their core to design novel hybrid bioinspired nanoparticles.[9,10] As such, the preparation of hybrid nanoparticles has been validated for a variety of inorganic cores, e.g., noble metals[11,12] or metal oxides,[13] and with different types of protein cages and virus-like particles, e.g., cowpea chlorotic mottle virus[14] or ferritins.[15–20] Interestingly, the development of protein cage-based hybrid nanoparticles[21] allowed one to combine into the hybrid material the physicochemical properties of the inorganic nanoparticles, e.g., plasmon resonance, fluorescence, with properties of biomaterials, e.g., biocompatibility. Therefore, such hybrid nanoparticles represent an opportunity for advanced nanotechnology applications in the fields of molecular imaging,[13,16,22] nano-electronics[23] and catalysis.[24,25]

A general concern in the synthesis of protein cage-based hybrid nanoparticles is the ability to control the interactions at the protein/inorganic nanoparticle interface.[26] Such control is necessary to achieve specific positioning of inorganic nanoparticles inside the protein cages’ core. Currently, two main approaches are used to synthesise such hybrid biomaterials. The first strategy uses a process called ‘encapsidation’ that is driven by the reversible self-assembly of a protein cage. Once disassembled, a mixture of protein subunits is present during the colloidal suspension of inorganic nanoparticles. Environmental changes, such as pH or temperature, are then used to trigger the reassembly of the protein cages. The self-assembling of protein cages occurring around the inorganic nanoparticles is directed by the affinity between the nanoparticles surface and the proteins, e.g., *via* electrostatic interactions.[17,26–28] The second approach was inspired by a biological process called ‘biomineralisation.’[14] Biomineralisation is a biochemical reaction occurring in living organisms to isolate, store and transport free ions, that are otherwise toxic.[29] A typical instance of a protein cage possessing this native biological function is ferritin. Ferritin is present in most living organisms and contains peptide sequences that facilitate the oxidation of iron ions and subsequent nucleation and rapid growth of an iron oxide nanoparticle in the ferritin core.[30] In this approach, the presence of pores in the protein cage structure is necessary to allow for the passage of surrounding metal ions into the core.

The unique feature of porous protein cages which allow for the internalisation of materials *via* nanometre size pores presents an attractive opportunity also for the internalisation of inorganic nanoparticles. Direct internalisation of nanoparticles inside porous protein cages could provide an alternative approach for the preparation of hybrid nanoparticles. However, to our knowledge this strategy remains largely unexplored. Ishii et al. published the first instance of internalisation of non-coated inorganic nanoparticle, i.e., quantum dots, into preformed tubular chaperonin proteins GroEL (from *Escherichia coli*) and T.th (from *Thermus thermophilus* HB8).[31] The study demonstrated the use of protein cages to enhance the optical and thermal stability of quantum dots. Following this research, other studies showed the use of electrostatic interactions from positively charged residues, e.g., histidine, lysine, to direct the assembly of uncoated water-soluble quantum dots to the external surface of protein cages[32,33] or into their core.[34] Direct internalisation of preformed inorganic nanoparticles is challenged by some important barriers. The inorganic nanoparticles must be smaller than the protein cage’s pores to be able to access the core. Such pores are typically up to a few nanometres in diameter.[1] In addition, non-specific interactions between the inorganic nanoparticles and the proteins must be also prevented to favour the internalisation of the nanoparticle into the core and to avoid the adsorption onto protein cages’ outer surface. The surface of the inorganic nanoparticles should therefore include an additional coating that prevents undesired interactions. Anti-fouling coatings made of long polymers such as polyethylene glycols were shown to be effective passivating materials and are commonly used in nanomedicine to prevent protein adhesion.[35] However, such polymer coatings increase significantly the nanoparticles size and hence limit the nanoparticles access to the protein cage’s core. Finally, the nanoparticles transport through the protein cage’s pores may be reversible. An additional affinity mechanism between the inorganic nanoparticle and the protein cage’s core is required to favour the internalisation process to particle escape.

In this investigation, we evaluate the specific internalisation *via* affinity binding of single small gold nanoparticles into porous protein cages. As a porous protein cage, we used the E2 protein cage derived from the *Geobacillus stearothermophilus* that presents 12 pores of 6 nm and an empty core of 13 nm in diameter. We engineered the E2 protein by site-directed mutagenesis with oligohistidine sequences of 4 different repeats exposed in the cages’ core. Dynamic light scattering and electron microscopy analysis confirmed well-conserved structure of the E2 protein cages mutated with bis- or penta-histidine sequences. To facilitate the internalisation, and to prevent non-specific binding of 3.9 nm diameter gold nanoparticles, their surface was passivated with a self-assembled monolayer (SAM) made of a mixture of short peptidols and thiolated alkane ethylene glycol ligands.[36,37] The functionalisation of the SAM-coated gold nanoparticles with Ni^2+^ nitrilotriacetic (Ni-NTA) moieties enabled the specific binding to oligohistidine tagged cages. Finally, the internalisation *via* affinity binding of Ni-NTA-functionalised peptide coated gold nanoparticles into the core of E2 protein cages was evaluated for the different E2 mutants and wild-type E2 and validated by electron microscopy analysis.

## Materials and Methods

### 2.1 Materials

The peptidol H-CVVVT-ol was purchased from Peptide and Protein Research (PPR Ltd., Hampshire, UK). HS-(CH_2_)_11_-EG_3_-NTA and HS-(CH_2_)_11_-EG_4_ ligands were purchased from Prochimia (Prochimia Surfaces Sp. z o.o., Sopot, Poland). Gold nanoparticles of 3.9 nm diameter stabilised in citrate buffer were purchased from Nanopartz Inc. (Canada). PBS buffer, Sephadex G25 superfine resin, dimethylsulfoxide (DMSO), Tween 20, nickel (II) nitrate hexahydrate and sodium phosphotungstate tribasic hydrate were purchased from Sigma-Aldrich Ltd. (Dorset, UK). Nanosep filters 10 kDa cut-off were purchased from PALL (PALL Corp., Portsmouth, Hants, UK). Affi-Gel 10 resin and Tris-HCl were obtained from Bio-Rad Laboratories Pte Ltd (Singapore). UV spectra were acquired with a Spectra Max Plus spectrophotometer (Molecular Devices, Wokingham, UK) using a 384 well plates from Corning (Lowell, US). *E*. *coli* strains DH5α (Zymo Research) and BL21(DE3) (Stratagene) were used as host cells. The vector pET-11a was purchased from Novagen. The oligonucleotides were synthesised by 1^st^ BASE (Singapore). Restriction enzymes (BamH I and Nde I), T4 DNA ligase, and Pfu Ultra High-Fidelity DNA polymerase, and isopropyl β-D-thiogalactopyranoside (IPTG) were obtained from Fermentas.

### 2.2 Preparation of NTA-functionalised peptide coated gold nanoparticles

A 2 mM solution of H-CVVVT-ol was prepared in DMSO:H_2_O milliQ 25:75 (v:v). Solutions of HS-(CH_2_)_11_-EG_3_-NTA and HS-(CH_2_)_11_-EG_4_ (both 2 mM) were prepared in DMSO and ethanol respectively. To prepare coated gold nanoparticles, 1 volume of 2 mM solution containing 60% of H-CVVVT-ol, 30% of HS-(CH_2_)_11_-EG_4_ and 10% HS-(CH_2_)_11_-EG_3_-NTA ligand was mixed with 1 volume of PBS 10X (81 mM Na_2_HPO_4_, 12 mM KH_2_PO_4_, 1.4 M NaCl, and 27 mM KCl, pH 7.4) and 0.005 volume 1% (v:v) Tween 20 in milliQ H_2_O. Then, 9 volumes of colloidal gold solution were added and mixed overnight at room temperature.

### 2.3 Purification of NTA-functionalised peptide coated gold nanoparticles by size exclusion chromatography

Sephadex G25 superfine (10 mL) was prepared, loaded into a column and kept in 0.02% (w:v) sodium azide. The column was equilibrated with 30 mL of 100 mM NaCl aqueous solution with 0.002% (v:v) Tween20. Coated gold nanoparticles (1.1 mL) were concentrated to approximately 150 μL by centrifugation on a 10 kDa cut-off Nanosep filter. Then, nanoparticles were loaded on the column and eluted under gravity. Approximately 1.4 mL coloured fractions corresponding to nanoparticles were collected in the excluded volume.

### 2.4 Nickel ion loading of NTA-functionalised peptide coated gold nanoparticles

One-tenth volume of 2.8 M Ni(NO_3_)_2_.6H_2_O aqueous solution was added to 1 volume of purified coated gold nanoparticles in 100 mM NaCl with 0.005% (v:v) Tween20. The reaction was mixed overnight at room temperature. The Ni^2+^ excess, not chelated to the NTA groups, was removed by size exclusion chromatography on Sephadex G25. The purified nanoparticles were finally dispersed in PBS pH 8.0 with 0.005% (v:v) Tween 20.

### 2.5 Preparation of the hexa-histidine resin

Affi-Gel 10 resin slurry (2 mL) was washed with 10mL of cold (4°C) 1 mM HCl, and then twice with DMSO, to give, 1 mL of resin. To this 950 mL DMSO, and 50 mL H-CVVVTGGGHHHHHH-OH peptide (5 mM, in DMSO) were added. The reaction was stirred overnight at room temperature before adding 6 mL 100 mM Tris-HCl, pH 8.5 for 4 h to block unreacted N-hydroxysuccinimide groups. The resin was washed three times alternately with 50 mM sodium acetate, 0.5 M NaCl, pH 4.5, and then with 100 mM Tris-HCl, pH 8.5. The resin was stored at 4°C in 20% (v/v) ethanol/water until further use.

### 2.6 Design and construction of E2 protein

Mutant plasmids were generated using site-directed mutagenesis. Oligonucleotides in Table 1 were used to PCR-amplify the mutant plasmid upon pET-11a plasmid containing wild-type E2 gene (pE2-WT).

**Table 1:** Oligonucleotides for plasmid construction.

Protein cage code	Oligonucleotide for plasmid construction^a^
E2-LH2	C GAA AAG CCG ATC GTT CGT **CAT CAT** GAA ATC GTT GCT GCT CCG AT
E2-LH5	C GAA AAG CCG ATC GTT **CAT CAT CAT CAT CAT** ATC GTT GCT GCT CCG AT
E2-LH6	C GAA AAG CCG ATC GTT **CAT CAT CAT CAT CAT CAT** ATC GTT GCT GCT CCG AT
E2-H3LH3	AAG CCG ATC GTT **CAT CAT CAT** CGT GAC GGT GAA **CAT CAT CAT** ATC GTT GCT GCT

^a^Mutation and insertion sites are in bold.

To construct the expression mutant vectors, the vector pET-11a and the PCR products were digested with NdeI and BamHI restriction enzymes. Both vector and target E2 genes were purified by 0.8% (w/v) agarose gel electrophoresis and ligated with T4 ligase. The sequences of the mutant genes were confirmed by the DNA sequencing service provided by 1^st^ Base.

### 2.7 Gene expression and protein purification

Wild-type E2 (E2-WT) and mutant proteins were produced in *E*. *coli* strain BL21(DE3) following a previously described protocol.[38] The *E*. *coli* cells were cultured in LB medium supplemented with 100 μg/ml ampicillin. Gene expression was induced by addition of 1 mM IPTG at an optical density (OD_600_) between 0.6 and 0.8. Cells were harvested 3 h after induction by centrifugation at 4500 ×g for 40 min and stored at -80°C. Subsequently, the cells were suspended in HEPES buffer (25 mM HEPES, pH 8.0) and disrupted by ultra-sonication. The supernatant after centrifugation at 40000 ×g for 1 h was heat-treated at 72°C for 20 min to denature the majority of *E*. *coli* proteins. Re-centrifugation at 40000 ×g for 1 h was then used to remove the denatured proteins.

The supernatant after ultra-centrifugation was filtered and loaded onto an anion exchange chromatography column (HiPrep Q 16/10 Q FF, GE Healthcare), which had been equilibrated with HEPES buffer (pH 8.0) on an ÄKTA liquid chromatography system (GE Healthcare). The E2 protein was eluted with HEPES buffer containing 1 M NaCl at elution concentration gradient set over 5 column volumes. Fractions containing the pure E2 protein were pooled. The concentration of protein was determined with a Micro BCA protein assay kit (Pierce) using bovine serum albumin (BSA) as the standard.

### 2.8 Determination of the self-assembly state of E2 protein cages

The self-assembly of mutant E2 protein cages in 20 mM Tris pH 8.7 was characterised by dynamic light scattering (DLS) (Zetasizer nano ZS, Malvern, UK). DLS was used to assess the hydrodynamic diameters of the proteins (1 mg/ml) at 25°C with 5 min equilibration. Results were an average of 3 measurements.

### 2.9 Internalisation of Ni-NTA-functionalised peptide coated gold nanoparticles into E2 protein cages

All protein cages were washed twice in milliQ water on Nanosep 10 kDa filters and dispersed in 50 mM NaH_2_PO_4_, 0.5 M NaCl pH 8.0 (phosphate buffer). Final concentrations of the cages were about 1 μM.

In 500 μL centrifuge tubes, 50 μL H-CVVVT-ol:HS-(CH_2_)_11_-EG_4_:HS-(CH_2_)_11_-EG_3_-NTA 60:30:10 (mole/mole/mole) coated gold nanoparticles (3.9 nm) at 0.4 μM were mixed with 50 μL of E2 protein cages at 1 μM overnight at room temperature. Samples were then stored at 4°C for a few days and analysed by electron microscopy.

### 2.10 High-resolution transmission electron microscopy analysis

Gold nanoparticles were concentrated by ultrafiltration of stock solutions with Nanosep 30 kDa filters. Concentrated solutions were deposited on Ultrathin Carbon (< 3 nm) on Carbon Holey support film TEM grids (Ultrathin Carbon / Holey Support on 400 mesh, Pelco International, USA). Gold nanoparticles and protein cages were analysed with a Philips CM300 high resolution analytical TEM/STEM. Typically, protein cage mixtures were analysed at 0.1 mg/mL stained with 1% (w/v) sodium phosphotungstate tribasic hydrate. Obtained images were not digitally modified, such as, for example, to remove the carbon grids. The size determination of the gold nanoparticles was done with ImageJ v1.47e software using the macro ‘ParticleSizeAnalyzer’.

## Results and Discussion

### 3.1 Method for internalisation of gold nanoparticles into engineered porous protein cages

**Fig 1:**
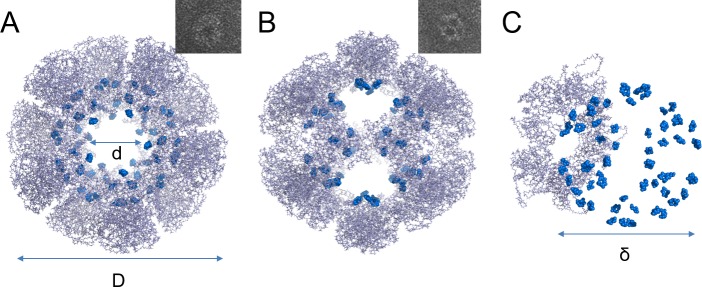
The E2 porous protein cage. (A) and (B) E2 protein cage three-dimensional structure (adapted from PDB ID: 1b5s).[42] The inserts show typical electron microscopy images of the 5-fold axis (A) and 2-fold axis (B) orientations of the protein cage E2. The diameter *D* of the E2 protein cage is 25 nm. The diameter *d* of each pore is 6 nm. (C) E2 protein cage inner surface presenting RDGE loop sequences in blue. The diameter δ of the cage core is 13 nm.

We used the E2 protein, the core unit of the pyruvate dehydrogenase multi-enzyme complex found in *Geobacillus stearothermophilus* as the porous protein cage model. The E2 protein cage consists of 60 identical subunits self-assembled into a dodecahedral protein complex.[38] The three-dimensional crystal structure of the E2 protein cage shows that it forms a cage-like structure of 25 nm in diameter and with 12 pores of 6 nm across (Fig 1). Because of its structural and physical properties, the E2 protein cage is an interesting template for various biological and chemical applications, and has been used as a platform for drug encapsulation[6,39] and therapeutic applications.[40,41]

**Fig 2:**
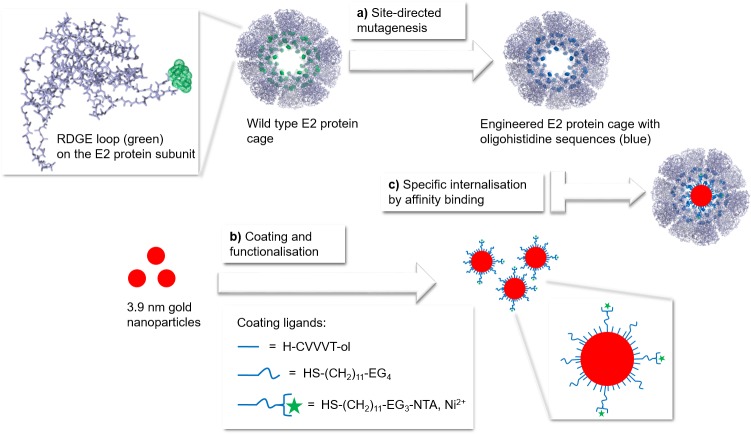
Method for specific internalisation of peptide coated gold nanoparticles into engineered E2 protein cages. (a) Site-directed mutagenesis of E2 protein cage’s core with oligohistidine sequences (blue) at the RDGE loop (green) on E2 protein subunits; (b) Surface coating of 3.9 nm gold nanoparticles with a self-assembled monolayer made of peptidols and thiolated alkane ethylene glycol (EG) ligands, functionalised with Ni^2+^ nitrilotriacetic moieties (NTA, Ni^2+^); (c) Specific internalisation by affinity binding of Ni-NTA-functionalised peptide coated gold nanoparticles into E2 protein cages presenting oligohistidine sequences.

The method proposed here is developed in three steps (Fig 2). We first introduced onto the inner surface of the protein cages oligohistidine sequences of various lengths by site-directed mutagenesis (Fig 2A). We chose to modify the E2 protein RDGE loop exposed in the cage’s core. To impart the affinity for the oligohistidine sequences present inside the E2 protein cage’s core, we passivated the surface of small gold nanoparticles with a SAM coating functionalised with Ni-NTA moieties (Fig 2B). The specific internalisation of coated gold nanoparticles inside mutated E2 protein cages (Fig 2C) was then monitored and evaluated by electron microscopy.

### 3.2 E2 protein cage platforms engineered with oligohistidine sequences

In this investigation, we considered two major factors that would drive the successful internalisation of small gold nanoparticles functionalised with Ni-NTA groups into oligohistidine mutated E2 protein cages: the stability of the protein cages and the binding strength between the oligohistidine sequences and the Ni-NTA groups.

We studied the formation of modified E2 protein cages presenting various oligohistidine sequences on the protein cages’ inner surface. To ensure the correct self-assembly and stability of the protein cages while providing sufficient access to the oligohistidine sequences, we chose to modify the RDGE loop present on the inner surface of the E2 protein cages. We synthesised four different variants of the E2 protein cage, i.e., E2-LH2, E2-LH5, E2-LH6 and E2-H3LH3, presenting an increasing number of histidine residues at the RDGE loop sequence by substitution and/or addition of histidine residues (Table 2). We evaluated each design for their ability to form stable protein cage self-assemblies and providing sufficient bonding strength between the oligohistidine sequences and the Ni-NTA groups to retain gold nanoparticles in their core. The E2-LH2 protein cage was modified the least and the length of the polypeptide backbone in the loop was unchanged, and therefore it was considered to have the least effect on the cage formation and might potentially possess sufficient affinity to retain Ni-NTA functionalised nanoparticles. In the case of the E2-LH5 protein cage, the polypeptide loop is longer by one amino acid and so it might affect the cage formation. Moreover, the five consecutive histidine’s side chains may also affect cage formation by driving unanticipated protein-protein interactions. This mutant was also more likely, compared to the E2-LH2 protein cage, to possess near full affinity similar to that of the standard hexa-histidine used in molecular biology/biotechnology. In fact, Knecht et al. demonstrated that oligohistidine sequences greater than four histidines provide highest binding affinity to Ni-NTA groups compared to smaller oligomers.[43] The E2-LH6 protein cage would possess full affinity to Ni-NTA groups, but would also have the greatest potential to affect the cage formation, since it had an increased length of the polypeptide backbone in the loop by two residues and the six consecutive histidine’s side chains may have some effect on the cage formation by driving unanticipated protein-protein interactions. Lastly, the E2-H3LH3 protein cage has the largest insert, with the histidines flanking the loop amino acids, which are retained. Splitting the hexa-histidine sequence with the core of the endogenous loop amino acids enabled to gain insight into the potential of histidine tracts of four or more in the loop to disrupt cage formation. Thus, this mutant allowed testing the idea that (within limits) the size of the loop can be substantially increased, but that four or more consecutive histidine residues (as in E2-LH5 and E2-LH6) are disruptive to cage formation.

**Table 2:** Dynamic light scattering analysis of wild-type and engineered E2 protein cages with different oligohistidine sequences at the RDGE loop.

Protein cage code	Protein sequence	Size (nm) peak 1^a^	Size (nm) peak 2^a^
E2-WT	. . . P^377^IVRDGEIVA^386^. . .	25.0 ± 0.6	-
E2-LH2	. . . P^377^IVR**HH**EIVA^386^. . .	25.3 ± 0.8	-
E2-LH5	. . .P^377^IV**HHHHH**IVA^387^. . .	26.1 ± 0.9	-
E2-LH6	. . .P^377^IV**HHHHHH**IVA^388^. . .	28.1 ± 1.2	-
E2-H3LH3	. . .P^377^IV**HHH**RDGE**HHH**IVA^392^. . .	24.3 ± 2.4	12.5 ± 0.8

^a^Averages of the size distribution by volume, obtained from three measurements for each protein cage.

**Fig 3:**
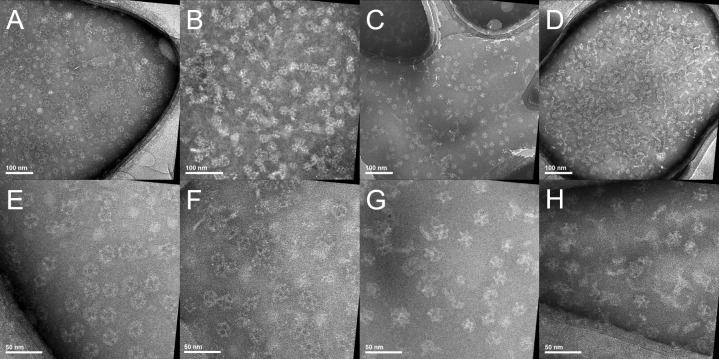
Electron microscopy of wild-type and engineered E2 protein cages. Images of the E2 protein cages: (A, E) E2-WT; (B, F) E2-LH2; (C, G) E2-LH5; (D, H) E2-LH6. (A–D) scale bars are 100 nm. (E–H) scale bars are 50 nm.

The correct formation and stability of the E2 protein cage mutants was confirmed by dynamic light scattering (DLS) measurement (Table 2) and by transmission electron microscopy (TEM) (Fig 3). The DLS size measurements showed that the introduction of bis-histidine (E2-LH2) and penta-histidine (E2-LH5) sequences per E2 protein subunit did not interfere with the correct formation of the E2 protein cages. However, the introduction of a hexa-histidine sequence resulted in a slightly increased size and the protein cages presented some instability during storage. The mutant E2-H3LH3 did not form a correctly assembled protein cages and was excluded from subsequent experiments. Hence, the results indicated that consecutive tracts of histidines are likely to be tolerated and that the size of the loop is probably the most important parameter to consider for the correct formation of stable protein cages.

### 3.3 Ni-NTA-functionalised peptide coated gold nanoparticles

**Fig 4:**
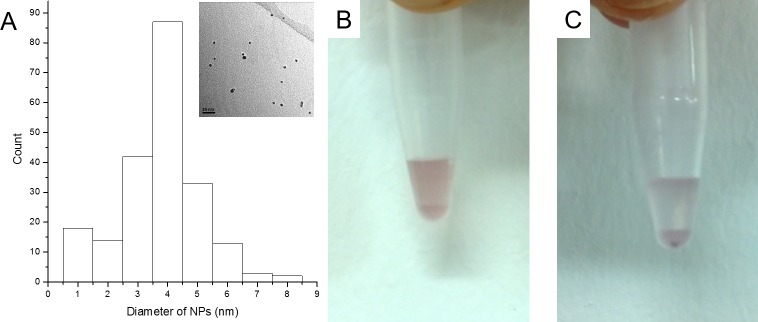
Ni-NTA-functionalised peptide coated gold nanoparticles. (A) Size distribution of 212 gold nanoparticles with an average diameter of 3.9 ± 0.8 nm. The insert shows a typical electron microscopy image used for size measurement. Immobilisation of Ni-NTA-functionalised peptide coated gold nanoparticles on a hexa-histidine loaded resin. (B) NTA-functionalised SAM coated gold nanoparticles not loaded with Ni^2+^ present no non-specific binding to hexa-histidine resin. (C) 10% (mol:mol) Ni-NTA-functionalised peptide coated gold nanoparticles fully bind to hexa-histidine resin as no free gold nanoparticles are found in the clear supernatant.

Gold nanoparticles of 3.9 nm in diameter (Fig 4A) were coated with multiple Ni-NTA groups. The preparation of NTA-functionalised peptide coated gold nanoparticles was done by passivation of citrate gold nanoparticles surface with a SAM consisting of a mixture of a peptidol H-CVVVT-ol and a thiolated alkane ethylene glycol ligand HS-(CH_2_)_11_-EG_4_ at a 60:30 ratio (mol:mol). The utilisation of such SAM has previously been shown to increase the stability[37,44] and facilitate the biofunctionalisation of gold nanoparticles.[36,45] An analogous SAM of pentapeptides have been shown to add 1.2 nm to the hydrodynamic radius of the nanoparticles.[46] NTA moieties were introduced to the monolayer by adding 10% (mol:mol) of the ligand HS-(CH_2_)_11_-EG_3_-NTA. The NTA-functionalised peptide coated gold nanoparticles were then loaded with Ni^2+^ for binding of the nanoparticles to oligohistidine sequences present on the inner surface of the protein cages. The functionalisation with HS-(CH_2_)_11_-EG_3_-NTA ligands and the specific binding to a oligohistidine tagged biomaterial were evaluated by the immobilisation of Ni-NTA-functionalised peptide coated gold nanoparticles on a hexa-histidine loaded agarose resin. All the Ni-NTA-functionalised peptide coated gold nanoparticles bound to the oligohistidine tagged resin, showing that 100% of the gold nanoparticles were functionalised with NTA moieties and activated with the Ni^2+^ (Fig 4C). Moreover, the experiment confirmed that in the absence of Ni^2+^ loading, the NTA-functionalised SAM coated gold nanoparticles did not present any non-specific binding to biomaterials (Fig 4B). This result corroborated some previously reported experiments where such SAM coated noble metal nanoparticles showed no non-specific binding to biomolecules and live cells.[37,44,45,47]

### 3.4 Peptide coated gold nanoparticles internalised into E2 protein cages

**Fig 5:**
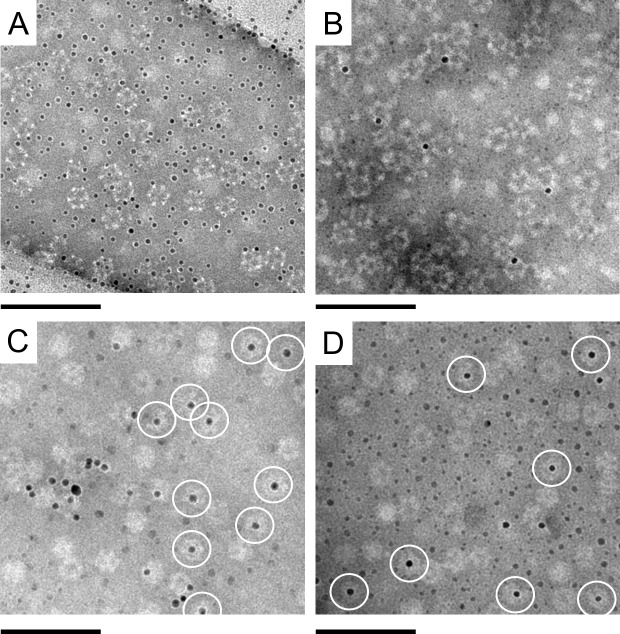
Transmission electron microscopy of samples of Ni-NTA-functionalised gold nanoparticles and E2 protein cages. Ni-NTA-functionalised gold nanoparticles mixed with (A) E2-WT, (B) E2-LH2, (C) E2-LH5, (D) E2-LH6. White circles indicate Ni-NTA-functionalised gold nanoparticles internalised into oligohistidine modified E2 protein cages. The samples were stained with 1% (w/v) phosphotungstic acid. Scale bars are 100 nm.

The internalisation of small Ni-NTA-functionalised gold nanoparticles into E2 protein cages was compared between the three mutated E2 protein cages, E2-LH2, E2-LH5 and E2-LH6, with the wild-type E2 protein cage as the control. The Ni-NTA-functionalised gold nanoparticles and the protein cages were washed in phosphate buffer at pH 8 and mixed with E2 protein cages at room temperature overnight. Each preparation was then analysed by TEM. The TEM images showed that for the E2-WT (Fig 5A) and the E2-LH2 (Fig 5B) there was no evidence for internalisation of the nanoparticles into the cages. In contrast, protein cages presenting longer oligohistidine sequences at their inner surface, i.e., E2-LH5 and E2-LH6, enabled the internalisation and immobilisation of Ni-NTA-functionalised gold nanoparticles. This was evidenced by the presence of multiple instances of gold nanoparticles perfectly aligned into the centre of the protein cages (white circles in Fig 5C and 5D). Such instances were not encountered in any analysis done with E2-WT or E2-LH2 protein cages. Hence a sequence of five or six histidines is needed to allow for the internalisation and subsequent immobilisation of Ni-NTA-functionalised gold nanoparticles. This result corroborates other studies that showed a greater binding strength between penta- and hexa-histidine sequences and Ni-NTA moieties compared to shorter oligohistidine sequences.[43] In addition, based on TEM, no obvious difference in the hybrid nanoparticle formation was observed between the E2-LH5 and E2-LH6 cages. Moreover, we also envisaged that the gold nanoparticles could potentially block the pores and remain in that position. In such case, more than one gold nanoparticle could attach to the protein cage at each of the 12 available pores. Based on TEM data, the binding of the gold nanoparticles was inside the lumen of the E2 protein cage (E2-LH5 and E2-LH6) and not at the pores. Also, since the pores are approximately 6 nm in diameter, we expected that only Ni-NTA-functionalised gold nanoparticles with sizes equal to or smaller than the pores will gain entry to the lumen. In addition, the lumen is only 13 nm and the longest distance of 8 nm between the loops.[48] Hence, as expected, we observed that only one nanoparticle can be encapsulated in each E2 protein cage. The number of hybrid nanoparticles observed by TEM seemed relatively low. This may be explained by the size of the coated gold nanoparticles used in this study that was close to the cage pores diameter of nearly 6 nm. Reduction of the diameter of the nanoparticles or the thickness of the ligand shell may be necessary to improve the internalisation efficiency and the purification of the hybrid nanoparticles from the excess of gold nanoparticles and empty protein cages (S1–S3 Figs) may be required for the further characterization and utilisation of the hybrid nanoparticles in various applications.

## Conclusions

We demonstrated the engineering of three novel porous E2 protein cages, i.e., E2-LH2, E2-LH5, E2-LH6, modified by site-directed mutagenesis and exposing bis-, penta- and hexa-histidine sequence into their core. Furthermore, the preparation of small Ni-NTA-functionalised gold nanoparticles passivated with a self-assembled monolayer of short peptidols and thiolated alkane ethylene glycol ligands allowed for their specific binding to hexa-histidine loaded agarose gels. The investigation of the combination of oligohistidine-modified porous E2 protein cages with Ni-NTA-functionalised peptide coated gold nanoparticles helped draw important conclusions on the feasibility of a direct and specific internalisation of inorganic nanoparticles into mutated E2 protein cages *via* affinity binding through the pores of the protein cages. The internalised gold nanoparticle will provide additional functionalities to the protein cage for future diagnostic development.

## Supporting Information

S1 Fig Purification of mixture of 3.9 nm diameter Ni^2+^ NTA-functionalised gold nanoparticles and 25 nm diameter Dyelight650 tagged E2-WT protein cages by size-exclusion chromatography. (PDF)

S2 Fig Purification of mixture of 3.9 nm diameter Ni^2+^ NTA-functionalised gold nanoparticles and 25 nm diameter E2-LH5 protein cages by size-exclusion chromatography. (PDF)

S3 Fig **Electron microscopy analysis of (A-B) the fraction 4 and (C-D) the fraction 6 after purification of mixture of 3.9 nm diameter Ni^2+^ NTA-functionalised gold nanoparticles and 25 nm diameter E2-LH5 protein cages by Superdex 200 size-exclusion chromatography.**
(PDF)
