# Supplementary material for: Specific Internalisation of Gold Nanoparticles into Engineered Porous Protein Cages via Affinity Binding
Source: PLoS One. 2016 Sep 13;11(9):e0162848. doi: 10.1371/journal.pone.0162848 (PMC5021291; doi:10.1371/journal.pone.0162848)
Supplement: S1 Fig — (PDF) [file pone.0162848.s001.pdf]

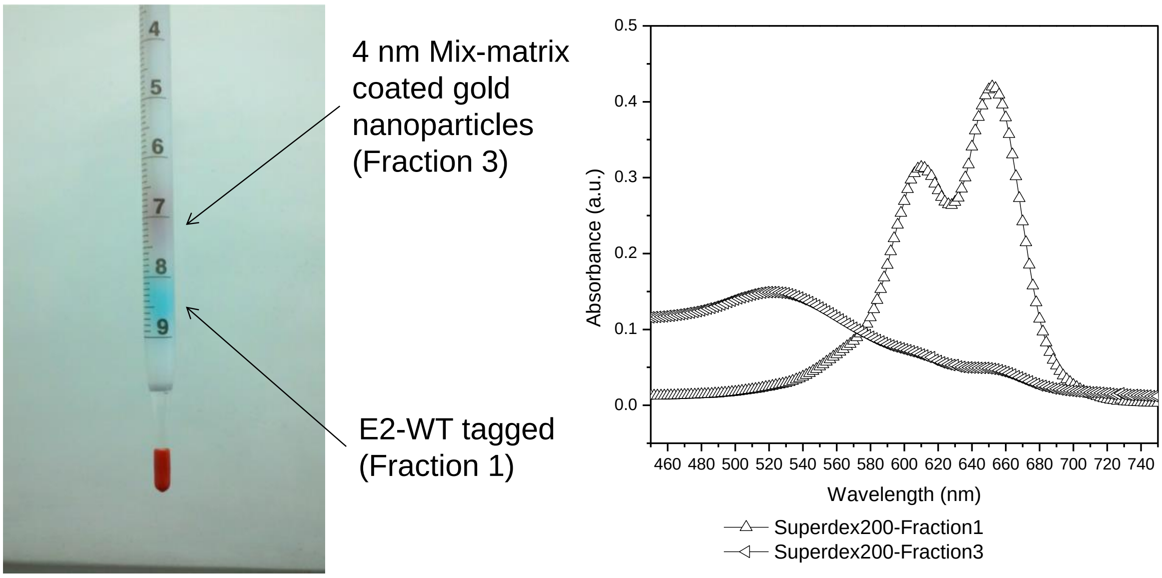

Figure S1. Purification of mixture of 3.9 nm diameter Ni^{2+} NTA-functionalised gold nanoparticles and 25 nm diameter Dyelight650 tagged E2-WT protein cages by size-exclusion chromatography. Left) Mixture of gold nanoparticles and tagged E2 protein cages are separated by Superdex 200 size exclusion chromatography on a 10 mL column, height 10 cm, diameter 1 cm at a flow of 1 mL/min. Right) UV-visible spectra of the two fractions containing the E2 protein cages (fraction 1) and the gold nanoparticles (fraction 3).
