# Supplementary material for: Specific Internalisation of Gold Nanoparticles into Engineered Porous Protein Cages via Affinity Binding
Source: PLoS One. 2016 Sep 13;11(9):e0162848. doi: 10.1371/journal.pone.0162848 (PMC5021291; doi:10.1371/journal.pone.0162848)
Supplement: S3 Fig — Electron microscopy analysis of (A-B) the fraction 4 and (C-D) the fraction 6 after purification of mixture of 3.9 nm diameter Ni2+ NTA-functionalised gold nanoparticles and 25 nm diameter E2-LH5 protein cages by Superdex 200 size-exclusion chromatography. (PDF) [file pone.0162848.s003.pdf]

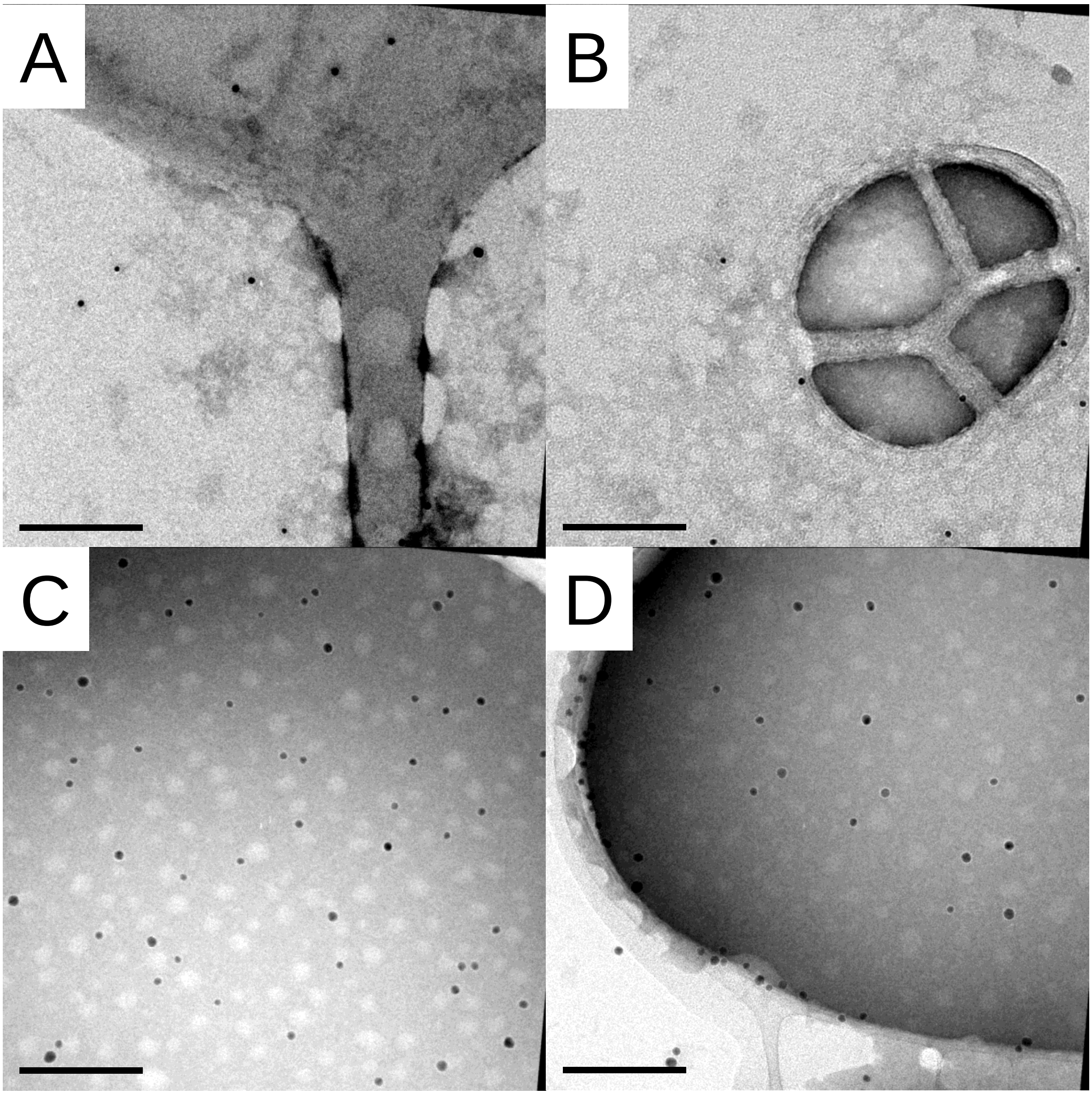

Figure S3. Electron microscopy analysis of (A-B) the fraction 4 and (C-D) the fraction 6 after purification of mixture of 3.9 nm diameter Ni^{2+} NTA-functionalised gold nanoparticles and 25 nm diameter E2-LH5 protein cages by Superdex 200 size-exclusion chromatography. The samples were stained with 1% (w/v) phosphotungstic acid. Both fractions show the presence of gold nanoparticles and protein cages.
